# Supplementary material for: ‘The Little Engine That Could’: A Qualitative Study of Medical Service Access and Effectiveness among Adolescent Athletics Athletes Competing at the Highest International Level
Source: Int J Environ Res Public Health. 2021 Jul 7;18(14):7278. doi: 10.3390/ijerph18147278 (PMC8304016; doi:10.3390/ijerph18147278)
Supplement: Supplementary file 1 [file ijerph-18-07278-s001.zip › ijerph-1242338-supplementary.pdf]

Qualitative study of Young Athletics Athletes' perception of Injury and Illness at the IAAF U18 World Championships in Nairobi, Kenya, on 12-16 July, 2017

Interview guide

Introduction 1 We are interested in getting a better understanding in how young Athletics athletes themselves perceive injury and illness. We therefore would like to ask you to think about episodes when a health problem, such as,
- pain, injury, soreness, a "feeling" (sensation)
- or illness symptoms (fever, nausea, sore throat)
caused you to change your planned training or competition program, or even completely abstain from athletics.

[PAUSE]

Introduction 2 Could you please think of the three most recent episodes when you made such changes?

[PAUSE]

Long episode (L) Out of these three episodes, please think about the episode when you were absent from athletics training and/or competition the longest period of time.

[PAUSE]

L1 How long ago did this episode occur (month/year)?

L2 Where were you at the start of the episode (training at home stadium, competing abroad, etc.)?

L3 What were you doing at the start of the episode (context: training, competing, etc.)?

[PAUSE]

L4.1 What are your perceptions of the general health problem leading up to this episode (body location, description of functional loss, symptom or "diagnosis", sudden/gradual onset, etc.)?

L4.2 Please describe to me as precisely as you can what it was that made you decide to change your planned training and competition.

L4.3 What do you believe caused the episode?

L4.4.1 What ensuing actions did you take in order to return to full training and competition after having decided to change your athletics routines?

L4.4.2 Did you find any of these actions being successful? If so, which and how?

L4.4.3 If not, why?

L4.5 When did you come back to full training with reference to the start of this episode?

L4.6 Can you remember what it was that made you decide to resume full training again? If so, please describe.

[PAUSE]

Short episode (S) Out of the three episodes, please now think about the episode when you were absent from athletics training and/or competition the shortest period of time.

[PAUSE]

S1 How long ago did this episode occur (month/year)?

S2 Where were you at the start of the episode (training at home stadium, competing abroad, etc.)?

S3 What were you doing at the start of the episode (context: training, competing, etc.)?

[PAUSE]

S4.1 What are your perceptions of the general health problem leading up to this episode (body location, description of functional loss, symptom or "diagnosis", sudden/gradual onset, etc.)?

S4.2 Please describe to me as precisely as you can what it was that made you decide to change your planned training and competition.

S4.3 What do you believe caused the episode?

S4.4.1 What ensuing actions did you take in order to return to full training and competition after having decided to change your athletics routines?

S4.4.2 Did you find any of these actions being successful? If so, which and how?

S4.4.3 If not, why?

S4.5 When did you come back to full training with reference to the start of this episode?

S4.6 Can you remember what it was that made you decide to resume full training again? If so, please describe.

[PAUSE]

Final F1. Do you have anything further that you would like to add that you believe would make it easier for us to understand how elite athletes perceive injury and illness?

Final F2. Was there anything associated to this interview that troubled you or caused inconvenience in association to your performance at the championships?

[PAUSE]

Personal (P) P1 What is your main event?

P2 What is your age?

P3 Athlete sex

P4 Athlete nationality
